# The Impact of *O*-Glycosylation on Cyanidin Interaction with RBCs and HMEC-1 Cells—Structure–Activity Relationships

**DOI:** 10.3390/ijms20081928

**Published:** 2019-04-19

**Authors:** Sylwia Cyboran-Mikołajczyk, Katarzyna Solarska-Ściuk, Katarzyna Mieszała, Natalia Glatzel-Plucińska, Karolina Matczak, Halina Kleszczyńska

**Affiliations:** 1 Department of Physics and Biophysics, Wroclaw University of Environmental and Life Sciences, Norwida 25, 50-375 Wrocław, Poland; katarzyna.solarska-sciuk@upwr.edu.pl (K.S.-Ś.); halina.kleszczynska@upwr.edu.pl (H.K.); 2 Department of Histology and Embryology, Medical University of Wroclaw, Chałubińskiego 6a, 50-368 Wrocław, Poland; katarzynamieszala@wp.pl (K.M.); n.m.glatzel@gmail.com (N.G.-P.); 3 Department of Medical Biophysics, University of Lodz, Pomorska 141/143, 90-236 Łódź, Poland; karolina.matczak@biol.uni.lodz.pl

**Keywords:** HMEC-1, anthocyanins, apoptosis, toxicity, SARs, intracellular ROS, RBCs

## Abstract

With the aim of contributing to the knowledge about their potential therapeutic activity, we determined the biological activities of cyanidin and its selected O-glycosides in relation to erythrocytes (RBCs) and human dermal vascular endothelial cells (HMEC-1). Furthermore, on the basis of changes in the physical/functional properties of the cells, the structure–activity relationships of the compounds were determined. Concerning erythrocytes, we analyzed the antioxidant activity of the compounds and their impact on the RBCs’ shape and transmembrane potential. The compounds’ cytotoxic activity, ability to modulate apoptosis, cell cycle, and intracellular ROS generation, as well as inhibitory activity against AAPH-inducted oxidative stress, were determined in relation to HMEC-1 cells. We demonstrated that biological activity of cyanidin and its *O*-glycosides strongly depends on the number and type of sugar substituents, and varies depending on the extracellular environment and type of cells. The compounds are practically non-cytotoxic, and do not induce apoptosis or disturb the progression of the cell cycle. Additionally, the compounds alter the shape of RBCs, but they do not affect their transmembrane potential. They effectively protect erythrocytes against free radicals and affect intracellular reactive oxygen spices (ROS) generation under physiological and AAPH-induced oxidative stress conditions. Our results suggest a potential beneficial effect of cyanidin on the cardiovascular system.

## 1. Introduction

Cyanidin and its glycosides occur practically in all colored plants, especially in fruit, vegetables, and flowers. Chemically, cyanidin as an aglycon (anthocyanidins) possesses two benzene rings, A and B, and a heterocyclic ring C, which form the C_6_-C_3_-C_6_ system. It contains five hydroxyl groups: At C_3_^′^ and C_4_^′^ positions of the B ring, C_7_ and C_5_ of the A ring, and at C_3_ of the heterocyclic C ring1 (Table 1). Glycosides (called anthocyanins) contain a smaller number of hydroxylated groups in the carbon skeleton (mostly four in mono-glycosides or three in di-glycosides) compared to aglycon, depending of the number of bonded sugars. The most common glycosides of cyanidin occurring in nature are *O*-glycosides, containing mainly glucose, galactose, arabinose, rhamnose, xylose, and homogeneous or heterogeneous disaccharides: Rutinose, sophorose, and sambubiose [1,2,3,4]. The 3-*O*-monosaccharides, 3-*O*-disaccharides, and 3-5-*O* and 3-7-*O* di-glucosides of cyanidin constitute about 50% of all phenolic pigments distributed in fruits and vegetables [5]. The wide presence of anthocyanins in nature and thus in the daily human diet results in a huge amount of research that has been focused on their potential health benefits [6]. It was shown that anthocyanins could effectively reduce the risk of atherosclerosis by improving endothelial dysfunction, inhibiting oxLDL formation, and promoting macrophage reverse cholesterol transport (RCT) [7,8,9]. They also may limit the risk of obesity and diabetes through, e.g., lowering the concentration of high-sensitivity C-reactive protein (CRP) and ameliorating disturbances in the lipid and glucose metabolism [10,11]. Anthocyanins possess neuroprotective activity related to their ability to induce autophagy [12], and anticancer activity mainly based on inhibition of the initiation, promotion, and progression of cancers, e.g., breast, liver, and blood [13,14,15]. The structure–activity relationship of both pure anthocyanins and that mixed in extracts has also been described in several works [6,16,17,18], but it still requires detailed studies. Especially, basic studies are needed in order to establish the molecular mechanism responsible for their beneficial effect on human health. Such an approach allows one to indicate the most effective substances in the prevention and treatment of many pathological conditions.

Cyanidin and its *O*-glycosides, like other anthocyanins, possess high biological activity, which has been demonstrated in numerous in vitro and in vivo studies. The most studied compound among cyanidin *O*-glycosides is cyanidin-3-*O*-glucoside, one of the most widely distributed [19]. It has been shown that cyanidin-3-*O*-arabinoside was effective in suppressing carrageenan-induced edema [20], and reduced superoxide production, neuron cell damage, lesion volume, and neurological dysfunction in a traumatic spinal cord injury (SCI) rat model [21]. It also suppressed the inflammatory response in rats by reducing the levels of inducible nitric oxide synthase (iNOS), tumor necrosis factor alpha (TNF-α), and the interleukins, IL-1β and IL-6, in peritoneal exudate cells [22] and inhibited the inflammatory response in adults with hypercholesterolemia [7]. Aglycone (cyanidin) inhibited lipopolysaccaride-induced cyclooxygenase 2 (COX-2) expression [23], influenced the function of endothelial cells, and exerted an inhibitory effect on oxidative injury of the cells [24]. Cyanidin also reduced intracellular ROS (reactive oxygen species) levels in the MCF-7, HuH-7, HepG2, Caco2, and HUVEC cell lines and inhibited the proliferation of cancer cells [25]. Cyanidin-3-*O*-rutinoside induced the accumulation of peroxides, which are involved in the induction of apoptosis in HL-60 cells [26] and may suppress the inflammatory response via inhibition of NO and PGE2 release and modulation of gene expression [27]. To the best of our knowledge, in the literature, there is a lack of studies determining and comparing the biological activity of cyanidin and its *O*-glycosides with respect to erythrocytes (RBCs) and immortalized human microvascular endothelial cells (HMEC-1 cells). Therefore, in this paper, using the example of cyanidin, the chemical features of anthocyanins (type of sugar, mono-, di-glycosides) have been placed in the context of their biological activity. The effect of cyanidin (C) and its selected glycosides (cyanidin-3-*O*-arabinoside (CA), cyanidin-3-*O*-glucoside (CG), cyanidin-3-*O*-galactoside (CGA), cyanidin-3-*O*-rutinoside (CR), and cyanidin-3-5-*O*-diglucoside (CDG)) on the physical and functional properties of cells was investigated. The cytotoxic, apoptotic, hemolytic, and antioxidant activity of the compounds was determined. Additionally, the impact of the compounds on the shape and transmembrane potential of erythrocytes as well as on the level of intracellular ROS and cell cycle progression was determined.

## 2. Results and Discussion

### 2.1. Cyanidin and its Glycosides Practically do not Induce Changes in Cell Viability, Cell Cycle, and Apoptosis of HMEC-1 Cells, by XTT, Hoechst, and Flow Cytometry Assays

**Figure 1:**
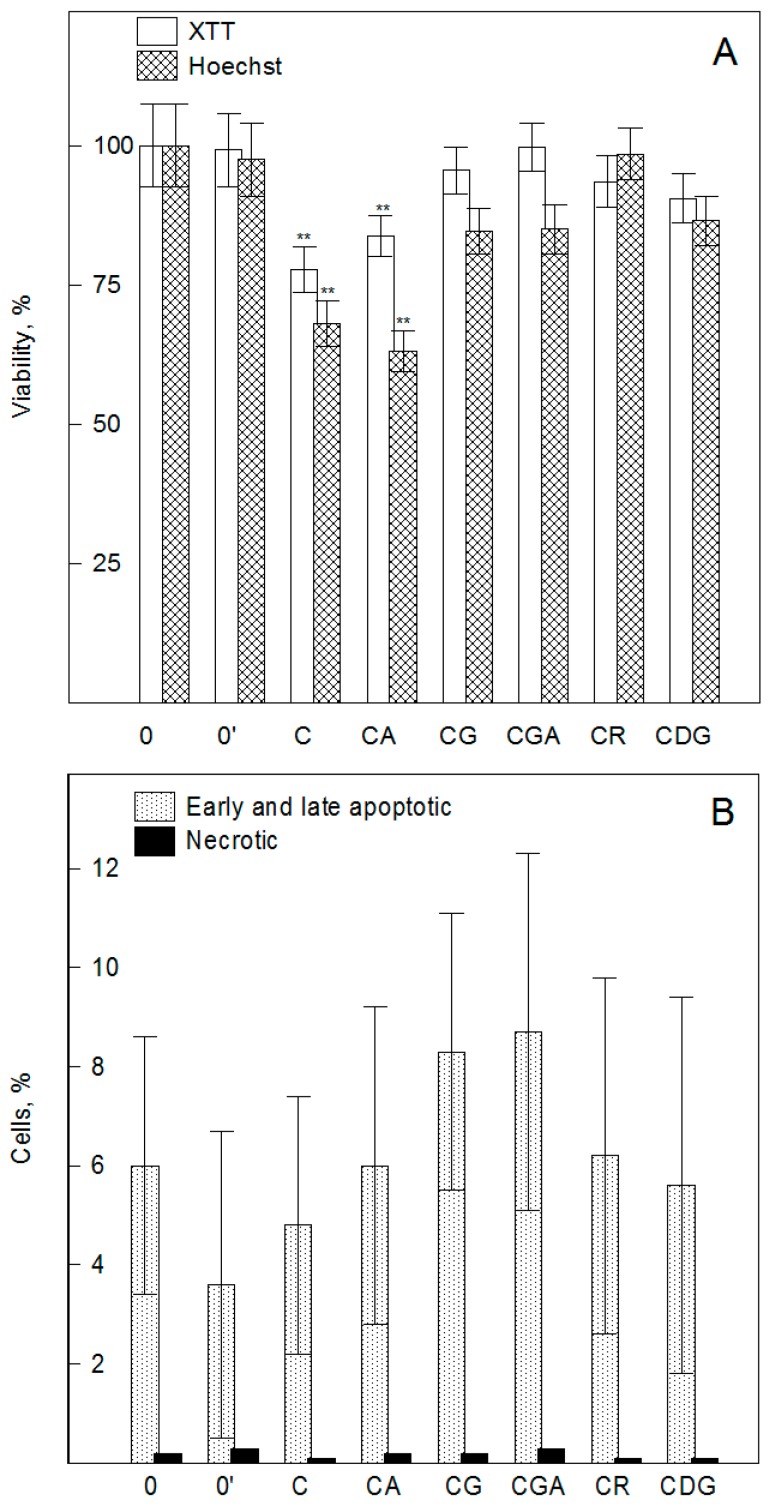

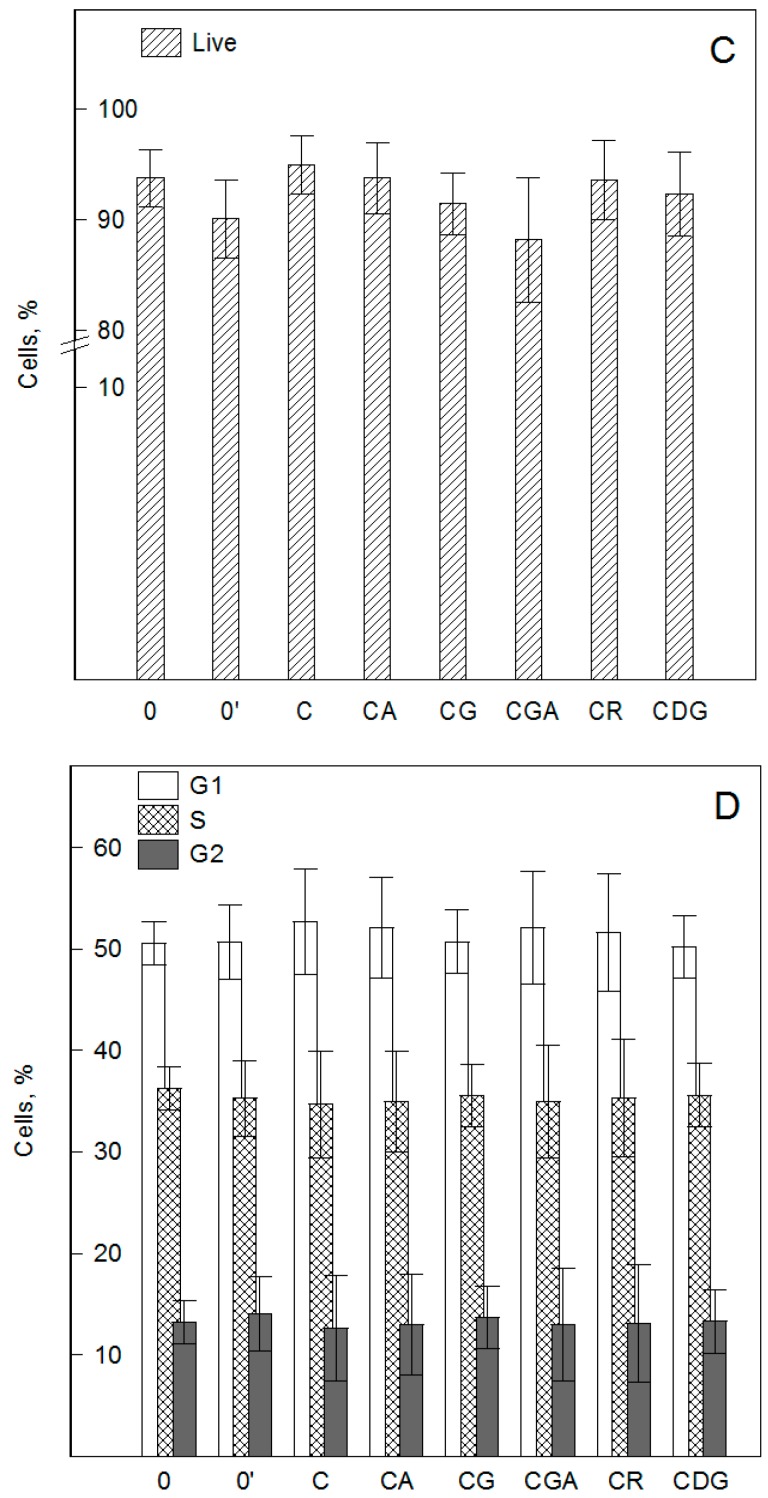
Effect of cyanidin and its glycosides on the viability of HMEC-1 cells determined using XTT (white bars) and Hoechst (crossed bars) assays (**A**). The ability of the compounds to induce apoptosis and cell cycle arrest: The percentage of early and late apoptotic and necrotic cells (**B**) and live cells (**C**) determined using flow cytometry assay of apoptosis, and the distribution of G1, S and G2, stages of the HMEC-1 cell cycle (**D**). The cells were incubated for 48 h with cyanidin and its glycosides at a concentration of 100 µM (viability assay) and at 50 µM (apoptosis and cell cycle flow cytometry assays). Cyanidin (C), cyanidin-3-*O*-arabinoside (CA), cyanidin-3-*O*-glucoside (CG), cyanidin-3-*O*-galactoside (CGA), cyanidin-3-*O*-rutinoside (CR), and cyanidin-3-5-*O*- diglucoside (CDG), control (0), and 0.5% ethanol-treated (0′) cells. Values are means ± SD (*n* = 3) of triplicate experiments. Statistically significant differences between the control and compound-modified cells are denoted as ^**^α = 0.01.

Prior to assessing the biological activity of the compounds and in order to assess their ability to inhibit cell viability, XTT (2,3-bis-(2-methoxy-4-nitro-5-sulfophenyl)-2H-tetrazolium-5-carboxanilide) and Hoechst 33324 (2,5′-bi-1H-benzimidazole, 2′-(4-ethoxyphenyl)-5-(4-methyl-1-piperazinyl)) assays were performed to exclude the possibility of cytotoxicity. Treatment of HMEC-1 cells with the compounds (10−100 µM) for 24 h did not induce any significant changes in cell viability (not shown). Forty-eight hours of cell modification with CG, CGA, CR, and CDG (10−100 µM) showed no cytotoxicity. C and CA (at 100 µM) significantly (*p* < 0.01) decreased the viability of endothelial cells compared with the control (Figure 1), as confirmed by both methods. The cell viability was decreased to approximately 65% to 80% and 60% to 85% (XTT and Hoechst assay) for C and CA, respectively, but there was no statistically significant difference between their effects. The inhibition of cell viability under the influence of the compounds was also observed by other authors in relation to a different cell line. Jung et al. [27] demonstrated that treatment of RAW 264.7 cells with CG and CR at 100 to 200 µg/mL significantly decreased the cell viability compared with control cells. A positive correlation (*r* = 0.776, *p* < 0.01) between the total content of hydroxyl groups in the molecules of anthocyanins/anthocyanidins and the survival of endothelial cells (EA.hy926) was observed by Yi et al. [16]. Taking into account that anthocyanins can be absorbed directly into the human blood circulatory system, and additionally that their bioavailability is rather low (the maximum concentration in blood after oral administration is in the nM range) [28,29], our results indicate that side effects of their overconsumption are unlikely. We observed a relatively small decrease in cell viability after a very long time of their modification at a concentration of about 1000 times higher than physiological levels.

Some anthocyanins, as literature data indicate, may modulate the toxic activity of cytostatics. Their harmful effect, as exemplified by doxorubicin, is based on, e.g., ROS overproduction, initiation of cell apoptosis, and arrest of the cell cycle [30]. Polyphenols may increase their cytotoxic activity in relation to tumor cells (synergistic effect) or decrease it with respect to normal cells [31,32]. Therefore, we determined the ability of the compounds to inhibit the cytotoxicity of doxorubicin, one of the most popular chemotherapy drugs administered intravenously. For this purpose, the HMEC-1 cells were first pre-incubated with the compounds (10−100 µM, 24 h) and then treated with doxorubicin for 4 h. The results of the XTT assay showed that cyanidin and its *O*-glycosides do not affect the cytotoxicity of doxorubicin (Figure S1, Supplementary Materials). We did not observe any significant differences between the cytotoxicity of doxorubicin in control cells and those modified with the compounds. This means that the presence of the compounds at a concentration of 100 µM in the cell membrane did not influence the doxorubicin toxicity with respect to endothelial cells. Next, the apoptotic effects of the compounds and their ability to induce changes in cell cycle progression were determined. The capability of cyanidin and its *O*-glycosides to induce apoptotic death of HMEC-1 cells was investigated using FITC Annexin V and propidium iodide (PI) staining followed by flow cytometric analysis. The representative histograms are shown in Figure S2. Cells’ treatment with 50 µM of the compounds for 48 h resulted in a lack of changes in early and late apoptotic and necrotic cell populations in comparison to untreated cells (Figure 1A,B). Furthermore, the flow cytometry analysis of the cell cycle showed that the used compounds do not induce any changes in its progression (Figure 1C). A representative analysis of the cell cycle is shown in Figure S3. Untreated cells remained in a balanced distribution of the cell cycle, which was similar to those obtained for treated cells. It means that cyanidin and its *O*-glycosides do not change the HMEC-1 cell distribution in any of the phases, i.e., G1, S, and G2/M, of the cell cycle. The harmful effect of doxorubicin, as the literature data indicate, may be based on ROS overproduction, initiation of cell apoptosis, and G2/M phase cell cycle arrest [30]. Therefore, the increased/decreased cytotoxic effect of doxorubicin by polyphenols may result from their antioxidant/pro-oxidant activity and ability to modulate cell cycle progression and apoptosis. The ability of cyanidin, as well as other anthocyanins, to induce programmed death of the cells has been described in several reports in relation to both healthy and tumor cells. The inhibition of cell proliferation and increased apoptosis of MCF-7 breast cancer cells were induced by C [33]. Liu et al. [34] found, in their study on renal cell carcinoma, that the percentage of apoptotic cells gradually increased after treatment with cyanidin (for 48 h at 0, 25, 50, and 100 μM) in a concentration-independent manner. What is more, cyanidin was found to affect late apoptosis in a dose-dependent manner and the effect of cyanidin induced cell cycle arrest (most cells arrested mainly at the phase G1-M stage) independently of the concentration [34]. CR may induce apoptosis in HL-60 cells by the accumulation of peroxides and possess relatively low toxicity toward human peripheral blood mononuclear cells [26]. CG exerted its cytotoxic effect by inducing apoptosis in cancer cells—HER2 (breast cancer), GBM (glioblastoma), and human monocytic leukemia—and interrupting the G2-M phase of the cell cycle [35,36,37]. The lack of apoptotic activity and ability to modulate cell cycle progression determined in our study are in good agreement with the lack of effect of the compounds on the cytotoxicity of doxorubicin. Additionally, our results indicate that the decreased viability of HMEC-1 cells under the influence of C and CA is probably caused by their pro-oxidant action.

### 2.2. Cyanidin O-Glycosides Modulate Intracellular ROS Generation in Vascular Endothelial Cells in Physiological and Oxidative Stress Conditions—H_2_DCF-DA Assay

**Figure 2:**
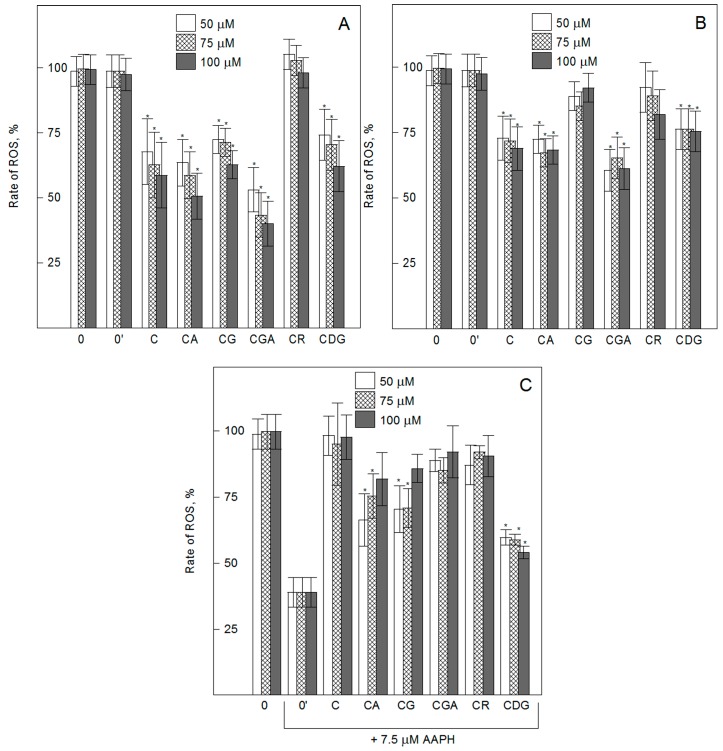
Intracellular reactive oxygen spices (ROS) generation in non-oxidized HMEC-1 cells (**A**,**B**) and AAPH-oxidized (**C**) treated with cyanidin and its glycosides used at concentrations from 50 to 100 µM. The non-oxidized cells were treated with the compounds for 24 h (**A**) and 48 h (**B**), oxidized cells were first pre-incubated with the compounds for 24 h and then incubated with AAPH for the next 24 h (**C**). The rate of ROS generation was calculated after 2 h of cells’ incubation with H_2_DCF-DA. Cyanidin (C), cyanidin-3-*O*-arabinoside (CA), cyanidin-3-*O*-glucoside (CG), cyanidin-3-*O*-galactoside (CGA), cyanidin-3-*O*-rutinoside (CR), cyanidin-3-5-*O*-diglucoside (CDG) treated cells, and control (0-non-oxidized, 0′-oxidized cells). Values are means ± SD of three independent measurements. Statistically significant differences between the control (or AAPH-oxidized) and compound-modified cells are denoted as ^**^α = 0.01.

In order to determine the ability of the compounds to modulate intracellular ROS generation, we used a H_2_DCF-DA (2′,7′-dichlorodihydrofluorescein diacetate) probe. A significant decrease in the intracellular ROS level was observed for cells treated with C, CA, CG, CGA, and CDG for 24 h, compared with control cells (Figure 2). CR treated cells did not differ in ROS generation from control cells. In addition, the results indicate that the ROS inhibitory activity of the compounds practically does not depend on their concentration (in the range of 50 to 100 µM), but is significantly lower with increased incubation time. After 48 h of cell modification, a decreased intracellular ROS level was observed for C, CA, CGA, and CDG treated cells. However, no significant difference was observed between the effects of either CR (50–100 µM) or CG (100 µM) and untreated control cells. The obtained results show that: 1) The small differences in the spectral structures (CGA and CG) may significantly alter the ROS inhibition of cyanidin 3-*O*-glycosides; 2) the presence of monosaccharide at C_3_ (CA, CGA, CG) does not change the inhibitory activity in comparison to aglycone (C), but the presence of disaccharides (CR) inhibits it significantly; and 3) the presence of an additional sugar group at C_5_ (CDG) does not change the inhibition of ROS generation determined for 3-*O*-glycosides of cyanidin. Contrary to our results, Takeuchi et al. [25] indicated that C decreased the intracellular ROS levels of different cell lines (MCF-7, HuH-7, HepG2, Caco-2, and HUVEC), but its *O*-glycosides (CG) did not, and therefore they postulated that these differences are not cell specific. On the other hand, similar to our results, decreased intracellular ROS levels under the influence of *O*-glycosides of cyanidin were observed, e.g., for human HaCaT keratinocytes and HUVEC cells [38,39]. The reasons for the discrepancies between the above mentioned results may be due to differences in the cell lines, concentrations of the compounds, assay conditions, or procedures used.

A few studies have shown the inhibitory effect of different anthocyanins on oxidative injury in endothelial cells [16,40]. This prompted us to undertake studies on the protective effects of the compounds under oxidative stress conditions. Therefore, in order to determine the intracellular radical scavenging activity of cyanidin and its *O*-glycosides, the HMEC-1 cells were treated with the oxidation inducer, AAPH (2,2′-azobis(2-amidinopropane) dihydrochloride). Free radicals created as a result of hemolytic decomposition of AAPH at 37 °C caused an increase in the ROS level by about 60% after 24 h of cells’ incubation with AAPH. A significant decrease in the intracellular ROS level in comparison with control AAPH-oxidized cells was observed for cells pretreated with 50 to 75 µM of CA, CG, and CDG (Figure 2). Additionally, the results showed that the presence of C and CR does not cause any inhibitory activity, whilst CDG showed strong inhibitory action throughout the used range of concentrations. For the highest concentration of 100 µM, only CDG showed a protective effect against AAPH-induced oxidative stress. These results indicate that 3-*O*-glycosylation and 3-5-di-glycosylation increase aglycone’s ability to scavenge intracellular ROS generated by AAPH. As in a previous study, it was also found that a slight difference in the spatial structure of the compounds, i.e., between CG and CGA, can significantly modulate the biological activity of the molecules. Our differences between the activity of aglycone and its 3-*O*-glycosides and between the *O*-glycosides are partially confirmed by the results of Yi et al. [16]. Therefore, we postulate that the differences between ROS inhibition are not only cell specific, but also depend on the mechanism of oxidative injury of endothelial cells.

### 2.3. Cyanidin and its O-Glycosides do not Induce Hemolysis of Erythrocytes, but Effectively Protect them against AAPH-Induced Oxidative Damage—Spectrophotometric Studies

**Figure 3:**
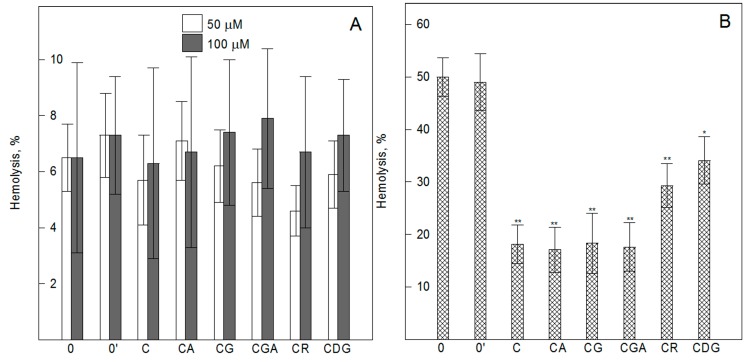
Percentage of hemolysis of erythrocytes: (**A**) Treated with 50 and 100 µM of cyanidin and its *O*-glycosides, (**B**) pre-treated with 8 µM of cyanidin and its glycosides for 1 h, and next treated with 120 mM of AAPH for 3 h. Cyanidin (C), cyanidin-3-0-arabinoside (CA), cyanidin-3-*O*-glucoside (CG), cyanidin-3-*O*-galactoside (CGA), cyanidin-3-0-rutinoside (CR), cyanidin-3-5-*O*-diglucoside (CDG) treated cells, control (0), and solvent control (0′). Values are mean ± SD of three independent measurements. Statistically significant differences between the control (or AAPH-oxidized) and compound-modified cells are denoted as ^**^α = 0.01, ^*^α = 0.05.

RBCs are the main cells in the circulation and are responsible for the transport of oxygen. They are characterized by a strong correlation between structure and biological function, which makes them a perfect model to study the interaction of different molecules with the organism at the cellular level [41,42]. The applicability of the in vitro cytotoxicity assay in RBCs is an alternative tool for the additional evaluation of compounds’ toxicity. Therefore, the hemolytic activity of the compounds was determined on the basis of the concentration of hemoglobin that was released from erythrocytes modified by cyanidin and its *O*-glycosides (5–100 µM) at 37 °C for 3 h. Our study showed that cyanidin and its *O*-glycosides do not cause leakage of hemoglobin from the cells even at a very high concentration of 100 µM (Figure 3A). The degree of their hemolytic activity evaluated using the mortality rate is in the range from 0% to 9% and indicates the lack of the compounds’ toxicity towards RBC.

Both the oxygen transport function and composition of the lipid membrane (mainly polyunsaturated fatty acids) mean that RBCs are constantly exposed to free radicals and are susceptible to their harmful effects. Therefore, the ability of the compounds to protect RBCs against oxidative damage induced by AAPH was examined. The results showed that the used compounds do not only induce hemolysis of RBCs, but they effectively protect them against water soluble free radicals (Figure 3B). This indicates that cyanidin and its *O*-glycosides possess a high ability to scavenge and neutralize AAPH-induced free radicals. The level of RBCs’ protection, provided by cyanidin, changes after its *O*-glycosylation. As the results show, it depends on the type and number of sugar substituents as follows: 3-*O*-glycosides containing monosaccharides (CA, CG, and CGA) indicate better protection than those containing disaccharides (CR) and two sugar substituents (CDG). We did not observe any significant differences between the antioxidant activity determined for C and its 3-*O*-glycosides with the attachment of monosaccharides (CG, CA, and CGA). Our results are in good agreement with literature data that also indicate the protective effect of selected cyanidin *O*-glycosides against free radical-induced damage of RBCs [43,44,45]. The relationship between the molecular structure and the antioxidant activity of anthocyanins is still not fully elucidated because, as literature data indicate, it depends on many factors, i.e., the type of anthocyanins tested, type of the free radicals, type of oxidized object, and the sensitivity of the method used [17,46]. Generally, glycosylation seems to decrease the antioxidant capacity of anthocyanins by reducing the number of free hydroxyls groups that are mainly responsible for ROS scavenging. It was shown that *O*-glycosylation of anthocyanins may (as in our case) either enhances or diminishes their antioxidant power depending on the anthocyanidin and lipid oxidation models used for antioxidant analysis [47]. Additionally, it was also shown that the smaller the number of sugar units at C_3_, the higher the antioxidant activity [47], and this finding is in agreement with our results. The better antioxidant activity of *O*-glycosides containing monosaccharides than those containing disaccharides was also reported by Jung et al. [27]. They found that CG had higher nitrite scavenging activity and a stronger protective effect against H_2_O_2_-induced cytotoxicity in macrophages than CR.

### 2.4. Cyanidin and its O-Glycosides Change the Shape of RBCs, but do not Alter their Transmembrane Potential—SEM and Fluorimetric Studies

**Figure 4:**
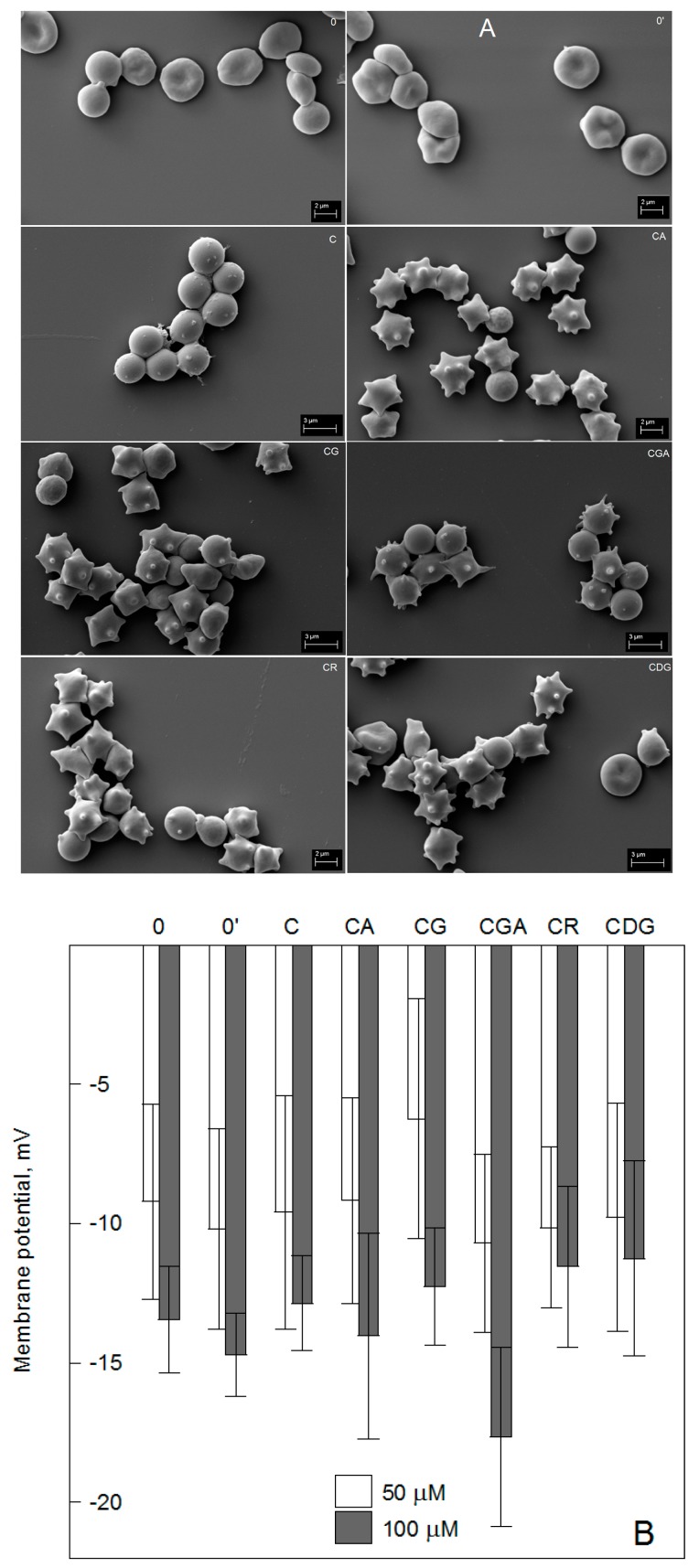
Representative photos of red blood cells treated with 100 µM of the compounds for 1 h (**A**). The red blood cells (RBC) photos were obtained using a scanning electron microscope and the experiment was performed three times. (**B**) The impact of the compounds on the transmembrane potential of erythrocytes was calculated with the Nernst equation. Values of the membrane potential are means ± SD (*n* = 3) of triplicate experiments. The RBCs were modified by 50 and 100 µM of the compounds. Cyanidin (C), cyanidin-3-*O*-arabinoside (CA), cyanidin-3-*O*-glucoside (CG), cyanidin-3-*O*-galactoside (CGA), cyanidin-3-*O*-rutinoside (CR), cyanidin-3-5-*O*-diglucoside (CDG) treated cells, control (0), and solvent control (0′).

The normal shape of RBCs is a flattened biconcave disc (discocyte) that may change under the influence of various factors, i.e., pH, pathogens, or exogenous bioactive substances. A good example of that is the treatment of erythrocytes in vitro with an amphipathic agent that causes RBCs’ transformation into various other shapes, such as echinocytes and stomatocytes. Such studies allow one to determine the location of exogenous substances in the RBC membrane. We studied the ability of C and its glycosides to induce changes in the shape of RBCs using a scanning electron microscope. A representative scanning electron image of RBCs treated with 100 µM of the compounds is shown in Figure 4A. It indicates that the compounds interact with the membrane and alter the normal biconcave morphology of the cells (Figure 4). Control erythrocytes and control-solvent (0.5% ethanol) treated erythrocytes were also assessed and found to be discoid or slightly echinocytic. Cyanidin and its glycosides induced the formation of echinocytes, but to a different degree. The largest changes in RBC shape were recorded for C, which caused the formation of the most advanced forms of echinocytes, i.e., spheroechinocytes, whose morphological index is +4, according to the Bessis scale [48]. Cyanidin *O*-glycosides containing glucose (CG) and galactose (CGA) showed similar effects and induced formation of mainly echinocytes of morphological indices of +2 and +3. CA also changed the RBC shape, but in this case, mainly less advanced forms of echinocytes, i.e., with an index of +2, were observed. The weakest ability to change shapes was exhibited by CR and CDG, which mainly caused the formation of discoechinocytes (+1). Echinocytic transformation is systematic and reversible and may occur in the presence of fatty acids, lysophospholipids, and amphiphatic drugs that are distributed preferentially in the outer lipid monolayer. According to the bilayer couple hypothesis, an increase of the surface area of the outer lipid monolayer relative to the inner leads to the formation of spicules [48,49]. Therefore, our results indicate that cyanidin and its *O*-glycosides bind to the RBC membrane and are located mainly in their outer lipid monolayer. Additionally, the demonstrated different ability to change the shape of RBCs suggests that cyanidin’s interaction with the erythrocyte membrane changes after *O*-glycosylation and depends on the type and number of the sugar substituents. Based on the observed shape transformation, we can conclude that C shows the greatest affinity for the membrane and it decreases less in the presence of glucose and galactose (CG and CGA) at C_3_ of the B ring than in the presence of arabinose (CA). The presence of disaccharide (CR) or two sugar substituents (CDG) significantly reduces the transformational ability of cyanidin and thus its binding to the RBC membrane. The echinocytic transformation of RBCs under the influence of different polyphenols was also observed by other authors [50,51]. It is considered that the echinocytic shape changes are the results of polyphenols interacting with lipids and the proteins of RBCs. Our earlier studies showed that cyanidin *O*-glycosides are located mainly in the area of the polar heads of membrane lipids, whilst C may be incorporated deeper into the membrane, becoming located on the border of the hydrophobic and hydrophilic area of the membrane. Their binding to the hydrophilic part of the outer lipid monolayer can increase the layer’s area and cause the formation of echinocytes. Additionally, after altering the physical properties of the lipid membrane, they can interact indirectly with the proteins of RBCs, which contribute to the maintenance of the shape, size, and elasticity of the cell [52]. Their presence in the hydrophilic part of the membrane suggests that they may also directly interact with the integral and cytoskeleton proteins of RBCs [51].

The observed RBC shape changes induced by the compounds indicate that they may also induce, directly or indirectly, changes in the transmembrane potential of the cell. The value of this potential reflects the potassium permeability of the erythrocyte membrane and for normal erythrocytes is within the range from −10 to −15 mV [53]. It depends mainly on the concentrations of ions within and outside the cell. Fluorimetric studies with a potential sensitive probe (DiSC_3_(5)) have shown that the used compounds do not change the transmembrane potential of RBCs (Figure 4B). We did not observe any statistically significant differences between the values of the transmembrane potential calculated for control and compound-modified RBCs. This signifies that the binding of the compounds to the RBC membrane does not alter ion permeation across the membrane.

## 3. Materials and Methods

### 3.1. Anthocyanins and Reagents

Cyanidin (C), cyanidin-3-*O*-glucoside (CG), cyanidin-3-*O*-galactoside (CGA), cyanidin-3-*O*-arabinoside (CA), cyanidin-3-*O*-rutinoside (CR), and cyanidin-3-5-diglucoside were purchased in Extrasynthese (Genay Cedax, France). Doxorubicin was obtained from Sequoia Research Products (Pangbourne, United Kingdom). The fluorescent probes, Hoechst 33342 nucleic acid, 3,3′-dipropylthiadicarbocyanine iodide (DiSC_3_(5)), 2′,7′-dichlorodihydrofluorescein diacetate (H_2_DCF-DA), and propidium iodide (PI), were purchased from Thermo Fisher Scientific, Inc., Waltham, Massachusetts, USA. Oxidation inducer 2,2′-azobis(2-amidinopropane) dihydrochloride (AAPH), 2,3-bis-(2-methoxy-4-nitro-5-sulfophenyl)-2H-tetrazolium-5-carboxanilide (XTT), Hank’s balanced salt solution (HBSS), antioxidant standard l(+) ascorbic acid (AA), and ionophore valinomycin were purchased from Sigma-Aldrich, Inc., Steinheim, Germany. FxCycle PI/RNase staining solution and the Dead Cell Apoptosis Kit with Annexin V FITC and PI were purchased from Thermo Fisher Scientific Inc., Waltham, MA, USA. All other reagents were analytically pure.

### 3.2. Erythrocytes and HMEC-1 Cells

The studies were conducted on pig erythrocytes and immortalized human microvascular endothelial cells (HMEC-1). The choice of pig erythrocytes was dictated by the fact that this cell’s percentage share of lipids is closest to that of the human erythrocyte, and the blood was easily available. The erythrocytes were obtained from fresh, heparinized pig blood. For washing the erythrocytes, an isotonic phosphate solution of pH 7.4 was used. The human dermal microvascular endothelial cell line, HMEC-1 (ATCC CRL 3243), was purchased from American Type Culture Collection (ATTC). The HMEC-1 cells were cultured in MCDB 131 medium containing 10% fetal bovine serum (FBS), 10 mM L-glutamine, 1 µg/mL hydrocortisone, 1% penicillin/streptomycin, and 10 ng/mL epidermal growth factor (EGF) purchased from Gibco or Sigma Aldrich, under 5% CO_2_ in plastic flasks at 37 °C. When the cells reached 80% confluence in the culture flasks, trypsin-EDTA was used to remove the cells. After trypsin neutralization with the medium, the cells were centrifuged (8 min, 800 rpm) and suspended in a small amount of medium. Next, the cells were counted using an automated cell counter, Countess (Invitrogen), and were used in experiments or reseeded in a flask.

### 3.3. Spectrophotometric and Fluorimetric Assays of the Viability of HMEC-1 Cells

The effect of compounds on the viability of HMEC-1 cells was assessed by XTT (2,3-bis-(2-methoxy-4-nitro-5-sulfophenyl)-2H-tetrazolium-5-carboxanilide) and Hoechst 33342 assays. Briefly, 5 × 10^3^ cells in 100 µL of the appropriate growth medium were plated in 96-well flat bottom clear microplates and cultured for 24 h at 37 °C in a humidified 5% CO_2_ incubator to ensure their logarithmic growth. Subsequently, medium was removed and 200 μL of the culture media containing various concentrations (10–100 μg/mL) of the compounds, dissolved in medium, were added to the wells and the cells were incubated for 24 h at 37 °C in a humidified 5% CO_2_ incubator. A corresponding volume of 0.5% ethanol was added to the control wells. In parallel experiments, some of the cells were additionally incubated with doxorubicin in a concentration of 0.5 µM (a cytotoxic compound usually used as an anticancer drug). After incubation, the medium in the wells was aspirated, the cell monolayers were rinsed twice with pre-warmed (37 °C) PBS, and the cells were allowed to grow for an additional 48 h. After incubation, during the XTT assay, medium was removed by aspiration and 50 µL of XTT/PMS solution (10 µL of 10 mM PMS + 4 mL of 4 mg/mL XTT solution) was added to cells. Cells were incubated in the dark for 2 h (37 °C, 5% CO_2_). After incubation, the absorbance of the samples was read at 450 nm and 690 nm using an Epoch Microplate Spectrophotometer, BioTek Instruments, Inc., Winooski, Vermont, USA. The fraction of surviving treated cells was presented as a percentage of viable control (untreated) cells taken as 100%.

In the Hoechst test, after incubation of cells with cyanidin and its glycosides, the medium was removed and the cells were washed twice with HBSS buffer containing 1% bovine serum albumin and frozen at −70 °C. Next, the cells were thawed at room temperature (RT) and re-frozen after the addition of 100 mL of distilled water. After thawing the plates at room temperature, 100 µL of HVAB solution (0.5% Hoechst in TNE solution containing 2 M NaCl, 1 mM EDTA, 10 mM Tris-HCl, pH 7.4) were added to each well. Next, cells were incubated for 15 min in the dark at 37 °C, mixed, and the fluorescence intensity of the probe was measured at 355 and 460 nm for excitation and emission, respectively (Fluoroskan Ascent FL, Thermo Scientific, Waltham, MA, USA). Cell viability was calculated as the ratio of the fluorescence intensity of the Hoechst 33342 probe for compound-modified to -unmodified cells and expressed as a percentage.

### 3.4. Flow Cytometry Assays of Apoptosis and the Cell Cycle of HMEC-1

The ability of the compounds to induce apoptosis and cell cycle arrest was studied using flow cytometry analysis. The cells (2.5 × 10^5^/well) were seeded in 6-well flat-bottom culture plates with 1 mL of culture medium and incubated for 24 h. Subsequently, medium was removed and 1 mL of culture medium containing 50 µM of the compounds, dissolved in medium, was added to the well. Then, the plates were incubated at 37 °C under 5% CO_2_ for 48 h. Next, the cells were carefully washed with 1 mL of PBS and 300 µL of trypsin was added and incubated for 3 min. After incubation, the trypsin was neutralized with 1 mL of the culture medium and the cells were collected and centrifuged (800 rpm, 5 min, RT). After that, the cells were washed twice with cold PBS and centrifuged (400× *g*, 7 min, RT). The cells were further diluted to a concentration of 1 × 10^6^ cells/mL and stained with an Annexin V FITC Dead Cell Apoptosis Kit, according to the manufacturer’s protocol. For each sample, data from a minimum of 10,000 events were collected. The results were further analyzed using FlowJo 10.5 software (FlowJo, Asham, OR, USA). For the cell cycle analysis, the cells were resuspended in a small amount of ice-cold PBS and fixed overnight at 4 °C in ice-cold 70% ethanol. Then, the cells were stained using FxCycle PI/RNase Staining Solution, according to the manufacturer’s protocol. After that, the samples were incubated for 30 min at 37 °C in the dark. The PI fluorescence was measured using a BD FACS Canto II flow cytometer (Becton Dickinson, Franklin Lakes, NJ, USA). For each sample, data from a minimum of 10,000 events were collected. The results were further analyzed using ModFit LT 5.0 software (Verity Software House, Topsham, ME, USA). The cell cycle analysis was performed in 3 independent replications, while the apoptosis assay was performed in 2 repetitions.

### 3.5. Fluorimetric Assay of Intracellular ROS Levels

The impact of investigated compounds on the level of intracellular reactive oxygen species and their ability to inhibit AAPH-stimulated intracellular ROS generation were assessed using a H_2_DCF-DA probe. Non-fluorescent H_2_DCF-DA is hydrolyzed intracellularly to form 2′,7′-dichlorodihydrofluorescein, which is oxidized by reactive oxygen species to the fluorescent 2′,7′-dichlorofluorescein. ROS production was estimated in HMEC-1 cells seeded on 96-well plates (5 × 10^3^/well) and cultured for 12 to 24 h. After that time, the compounds were added at appropriate concentrations. The incubation was continued for another 24 h and 48 h at 37 °C under 5% CO_2_. In the case of AAPH-simulated ROS generation, the cells were incubated with extracts for 24 h. After incubation, the AAPH dissolved in culture medium was added to each well at the final concentration of 7.5 mM and incubated for the next 24 h. After that time, the cell monolayers were rinsed with HBSS containing 1% albumin and 5 µM of the fluorescence probe, 2′,7′-dichlorodihydrofluorescein diacetate (H_2_DCF-DA), was added. Immediately and after 0.5 h, 1 h, and 2 h incubation in the dark in standard conditions, the fluorescence was measured at λ_ex_ = 485 nm and λ_em_ = 538 nm.

In the next step, DNA content was determined. For this purpose, after measurements, the cells were frozen at −70 °C. Next, the cells were thawed and re-frozen after the addition of 100 mL of distilled water/well. Then, the cells were thawed again. After that, 5 µM of RNase was added to each well and incubated for 30 min in the dark at 37 °C. Next, 50 µL of 2.5 µM propidium iodide was added to the cells and incubated for 5 min in the same conditions. The fluorescence intensity of PI was measured at λ_ex_ = 355 nm and λ_em_ = 620 nm. The rate of ROS generation was calculated according to the formula:

(1) $$Rate\;of\;ROS=\frac{IFe_{1}}{IFe_{2}}/\frac{IFc_{1}}{IFc_{2}}\cdot 100\%$$

where *IFe*_1_ and *IFe*_2_ are fluorescence intensities of H_2_DCF-DA and PI in compound-modified or compound-modified and AAPH-oxidized cells, and *IFc*_1_ and *IFc*_2_ are fluorescence intensities of H_2_DCF-DA and PI in control or AAPH-oxidized control cells.

### 3.6. Colorimetric Studies of Hemolytic and Antioxidant Activity

Upon removal from plasma, the erythrocytes (RBCs) were washed four times in phosphate solution (pH 7.4) and then incubated in the same solution, but containing appropriate amounts of the compounds studied. The modification was conducted at 37 °C for 1 h, each 1 mL sample containing an RBC suspension of 1.2% hematocrit and 1 to 100 µM of compounds, stirred continuously. After the modification, 2 mL of phosphate buffer were added and samples were centrifuged, and the supernatant assayed for hemoglobin content using a spectrophotometer (Specord 40, Analytik Jena) at the 540 nm wavelength. To test the effect of compounds on hemolysis inducted by AAPH, RBCs were pre-incubated with varying concentrations of compounds at 37 °C for 1 h. Hemolysis of RBCs was carried out by mixing 3% suspension of RBCs (unmodified or modified by 2 to 20 µM of the compounds) in phosphate buffer with AAPH solution (final concentration 120 mM). This reaction mixture was incubated for 3 h at 37 °C. After incubation, samples were centrifuged for 15 min (2000× *g*, RT). The extent of hemolysis was determined spectrophotometrically by measuring the absorbance of the supernatant at 540 nm. Hemoglobin concentration in the supernatant, expressed as a percentage of the hemoglobin concentration in the supernatant of totally hemolyzed cells, was assumed as the measure of the extent of hemolysis.

### 3.7. SEM Assay of the Shapes of Erythrocytes

The effect of compounds on the shape of erythrocytes was investigated using a scanning electron microscope (SEM). The red cells, when separated from plasma, were washed four times in saline solution and suspended in the same solution, but containing 50 µM of cyanidin or its *O*-glycosides. Hematocrit concentration of the erythrocytes in the modification solution was 2%, the modification lasting 1 h at 37 °C. After modification, the erythrocytes where fixed for 48 h in a 2.5% solution of glutaraldehyde. After that, the preparations were washed in phosphate buffer for 20 min, and then the material was dehydrated in a rising series of acetone concentrations (30%, 50%, 60%, 70%, 80%, 90%, and 100%). Each sample was washed for 15 min in an appropriate concentration, the material remaining in pure acetone for 30 min. Next, the erythrocytes were dried for 12 h at room temperature. Erythrocytes thus prepared were deposited on object stages and subjected to x-ray microanalysis by means of an x-ray analyzer, Bruker AXS Quantax, collaborating with the ESPRIT ver. 1.8.2. software. Next, the samples were coated with gold using a Scancoat 6 (Edwards, London) sprinkler. The material ultrastructure was analyzed using a scanning microscope (EVO LS15 ZEISS) with an SE1 detector, under high vacuum and accelerating voltage, acceleration voltage (EHT) = 20 kV. Individual forms of erythrocyte cells were ascribed morphological indices according to the Bessis scale [54], which for stomatocytes assume negative values from −1 to −4, and for echinocytes from 1 to 4.

### 3.8. Fluorimetric Assay of the Transmembrane Potential of RBCs

The transmembrane potential of erythrocytes was measured with the fluorescence indicator, DiSC_3_(5), according to a procedure described by Zavodnik et al. [55], with minor modifications. Briefly, 1 mL of erythrocytes solution of 2% hematocrit was incubated with increasing concentrations of compounds for 1 h at 37 °C. After this modification, 2 mL of 310 mOsm PBS was added to the suspension, centrifuged for 15 min at room temperature, and the supernatant removed, in order to separate the cells from the extract solution. Next, 220 µL samples were suspended in buffered saline, containing 10 mM Tris-HCl, pH 7.4, and 150 mM (KCl + NaCl), with the K^+^ concentration increasing from 50 to 140 mM. Next, an ethanolic solution of DiSC_3_(5) was added to a final concentration of 2 mM, and samples were incubated for 10 min at room temperature. The fluorescence intensity of the dye (I) was measured with a fluorimeter (CARY Eclipse of VARIAN) at 660 nm and excited at 625 nm. Then, the ionophore, valinomycin, was added to the samples to a final concentration of 1 mM, incubated for 10 min, and the fluorescence intensity of the probe (I_v_) was measured again. From the plotted dependence between (I-I_v_)/I and log_2_ of the external K^+^ concentration, the external potassium concentration ($K_{ex}^{+}$) for which no change in DiSC_3_(5) fluorescence intensity occurs upon valinomycin addition was calculated. The transmembrane potential (*E_m_*) was calculated from the Nernst equation for monovalent ions:

(2) $$E_{m}=\frac{R\cdot T}{F}ln\frac{K_{ex}^{+}}{K_{in}^{+}}$$

where *E_m_* is the transmembrane potential; and *T*, *R*, and *F* are the temperature, gas constant, and Faraday’s constant, respectively. $K_{ex}^{+}$ is the extracellular potassium concentration and $K_{in}^{+}$ is the intracellular potassium concentration of erythrocytes (152 mmol/L).

## 4. Conclusions

Our study has shown that cyanidin exhibits high biological activity in relation to erythrocytes and vascular endothelial cells. That activity changes significantly after *O*-glycosylation and depends on many factors, e.g., type and number of sugar substituents, type of cells, and experimental conditions. On the basis of toxicity assays, we can conclude that side effects of cyanidin overconsumption are unlikely and the use of the compounds at concentrations at which they exhibit biological/therapeutic activity is safe for vascular endothelial cells and erythrocytes. Furthermore, the ability of the compounds to modulate intracellular ROS generation under physiological and oxidative stress conditions and their antioxidant protection of cells make them useful for health care applications. In particular, as nutraceutical food additives, they may improve the quality and sensory properties of foods. Additionally, they can be used in the prevention and treatment of cardiovascular diseases, especially those associated with an imbalance between oxidizing and reducing agents.

## Figures and Tables

**Table 1:** Chemical structures and molecular characteristics of cyanidin and its *O*-glycosides.

Abbreviation	Substitution pattern		Structure
	R_1_	R_2_	
**C**	H	H	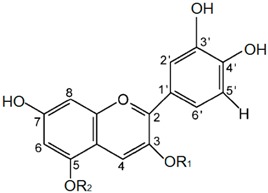
**CG**	glucose	H	
**CR**	rutinose	H	
**CGA**	galactose	H	
**CDG**	glucose	glucose	
**CA**	arabinose	H	

## Supplementary Materials

Supplementary materials can be found at https://www.mdpi.com/1422-0067/20/8/1928/s1.
