# Supplementary material for: The Impact of O-Glycosylation on Cyanidin Interaction with RBCs and HMEC-1 Cells—Structure–Activity Relationships
Source: Int J Mol Sci. 2019 Apr 19;20(8):1928. doi: 10.3390/ijms20081928 (PMC6514962; doi:10.3390/ijms20081928)
Supplement: Supplementary file 1 [file ijms-20-01928-s001.pdf]

The impact of O-glycosylation on cyanidin interaction with erythrocytes and HMEC-1 cells. SARs - structure-activity relationship

Sylwia Cyboran-Mikołajczyk[1]*, Katarzyna Solarska-Ściuk[1], Katarzyna Mieszała[2], Natalia Glatzel-Plucińska[2], Karolina Matczak[3], Halina Kleszczyńska[1]

[1]Department of Physics and Biophysics, Wrocław University of Environmental and Life Sciences, Norwida 25, 50-375 Wrocław, Poland,

[2]Department of Histology and Embryology, Medical University of Wroclaw, Chałubińskiego 6a, 50-368 Wrocław, Poland

[3]Department of Medicinal Biophysics, University of Lodz, Pomorska 141/143,

90-236 Łódź, Poland

*Corresponding author: tel. +48713205275, fax. +48713205167, e-mail: sylwia.cyboran@upwr.edu.pl, address: Department of Physics and Biophysics, Wroclaw University of Environmental and Life Sciences, Norwida 25, 50-375 Wrocław, Poland,

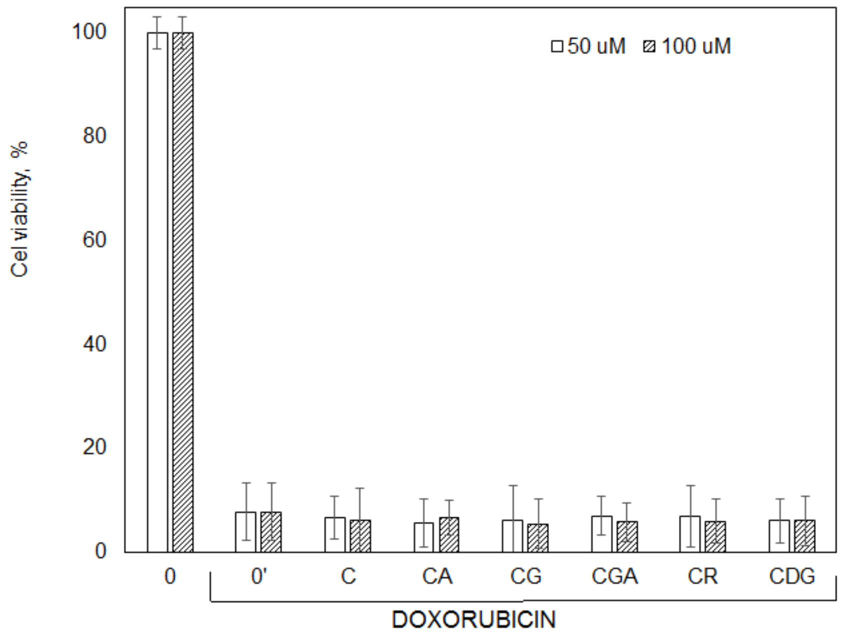

Fig. 1S The viability of HMEC-1 cells pre-treated with cyanidin and its O-glycosides for 24 h and treated with doxorubicin for next 24. Cells variability was determined using XTT assay. Cyanidin (C), cyanidin-3-*O*-arabinoside (CA), cyanidin-3-*O*-glucoside (CG) cyanidin-3-*O*-rutinoside (CR) and cyanidin-3-5-*O*-diglucoside (CDG), control (0), ethanol-treated cells (0’).

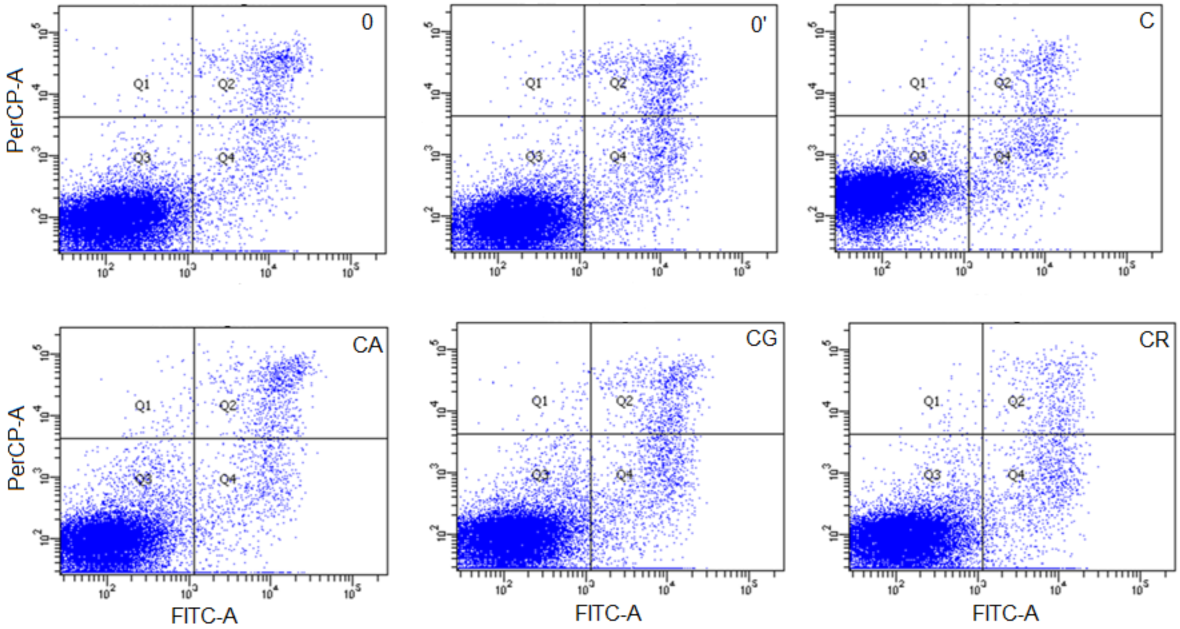

Fig. 2S An example representative flow cytometric analysis of cyanidin and its glycosides-treated HMEC-1 cells. Cells were stained with FITC Annexin V and propidium iodide (PI) after treatment with 50 µM of the compounds for 48 h. The graphs show the number of live (Q_3), early apoptotic (Q_4), late apoptotic (Q_2) and necrotic (Q_1) stages of cell cycle. Cyanidin (C), cyanidin-3-*O*-arabinoside (CA), cyanidin-3-*O*-glucoside (CG) cyanidin-3-*O*-rutinoside (CR) and cyanidin-3-5-*O*-diglucoside (CDG), control (0), ethanol-treated cells (0’).

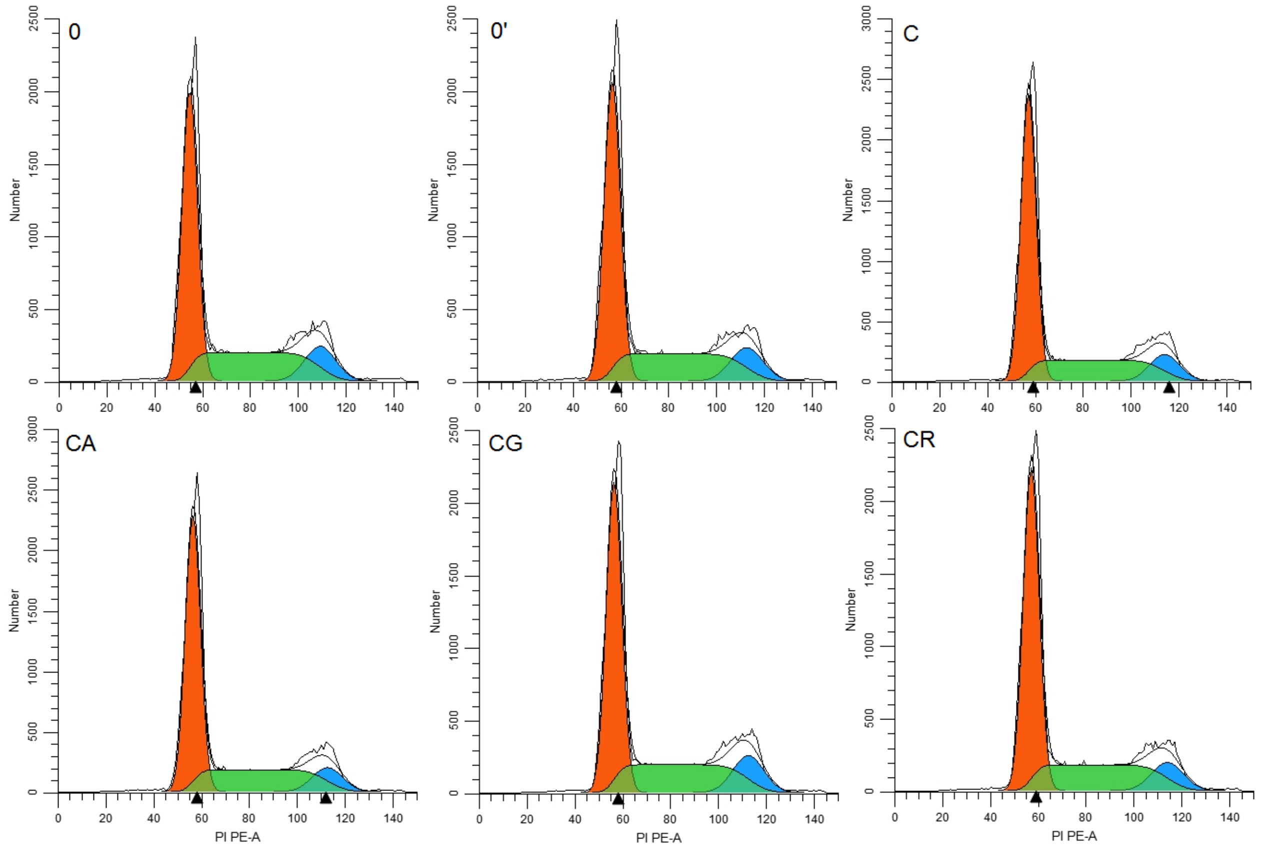

Fig. 3S An example representative flow cytometric analysis of cyanidin and its glycosides-treated HMEC-1 cells. Cells were stained with propodium iodide after treatment with 50 µM of the compounds for 48 h. The histograms show the number of cells in G_1 (red), S (green) and G_2/M (blue) stages of cell cycle, respectively. Cyanidin (C), cyanidin-3-*O*-arabinoside (CA), cyanidin-3-*O*-glucoside (CG) cyanidin-3-*O*-rutinoside (CR) and cyanidin-3-5-*O*-diglucoside (CDG), control (0), ethanol-treated cells (0’).
